# Activation of the endoplasmic reticulum stress sensor IRE1α by the vaccine adjuvant AS03 contributes to its immunostimulatory properties

**DOI:** 10.1038/s41541-018-0058-4

**Published:** 2018-06-28

**Authors:** Charlotte Givord, Iain Welsby, Sophie Detienne, Séverine Thomas, Assiya Assabban, Viviana Lima Silva, Céline Molle, Romain Gineste, Marjorie Vermeersch, David Perez-Morga, Oberdan Leo, Catherine Collignon, Arnaud M. Didierlaurent, Stanislas Goriely

**Affiliations:** 1 Institute for Medical Immunology, Université Libre de Bruxelles (ULB), Gosselies, Belgium; 2 GSK, Rixensart, Belgium; 3 Laboratory of Molecular Parasitology, IBMM, ULB, 12 rue des Prof. Jeener et Brachet, B-6041 Gosselies, Belgium; 4 Center for Microscopy and Molecular Imaging (CMMI), ULB, 8 rue Adrienne Bolland, B-6041 Gosselies, Belgium; 5 Laboratory of Immunobiology, Department of Molecular Biology, ULB, Gosselies, Belgium

## Abstract

The oil-in-water emulsion Adjuvant System 03 (AS03) is one of the few adjuvants used in licensed vaccines. Previous work indicates that AS03 induces a local and transient inflammatory response that contributes to its adjuvant effect. However, the molecular mechanisms involved in its immunostimulatory properties are ill-defined. Upon intramuscular injection in mice, AS03 elicited a rapid and transient downregulation of lipid metabolism-related genes in the draining lymph node. In vitro, these modifications were associated with profound changes in lipid composition, alteration of endoplasmic reticulum (ER) morphology and activation of the unfolded protein response pathway. In vivo, treatment with a chemical chaperone or deletion of the ER stress sensor kinase IRE1α in myeloid cells decreased AS03-induced cytokine production and its capacity to elicit high affinity antigen-specific antibodies. In summary, our results indicate that IRE1α is a sensor for the metabolic changes induced by AS03 in monocytic cells and may constitute a canonical pathway that could be exploited for the design of novel vaccine adjuvants.

## Adjuvants: ER stress is critical for adjuvant efficacy

Adjuvants are the ‘secret sauce’ of vaccines—by stimulating the innate arm of the immune system they potentiate activation of adaptive immunity; however, their mode of action is incompletely understood. A team led by Stanislas Goriely at the Université Libre de Bruxelles studied the functional basis of a major class of clinically-applied adjuvant—AS03—an oil-in-water emulsion adjuvant used in human influenza vaccines. Using an in vivo mouse model and cell lines they observe that AS03 triggered downregulation of genes controlling lipid metabolism. At the same time there was an increase in an ER-stress signature likely as a result of accumulation of intracellular lipid content in myeloid cells such as macrophages. ER stress was critical for the production of inflammatory cytokines by macrophages and the subsequent generation of robust and specific antibody responses following vaccination. Selective enhancement of ER stress might therefore lead to superior vaccines while minimizing side effects.

## Introduction

One of the general mechanisms through which adjuvants enhance adaptive responses to vaccine antigens is the rapid activation of the innate immune system.^1^ A handful of adjuvants are currently used in licensed human vaccines. Some of them, such as monophosphoryl lipid A (MPL), act directly through a well-defined pathogen recognition receptor. For other adjuvants including Alum, saponins or oil-in-water emulsions, specific receptors are not defined and activation of innate responses involves the extracellular release of endogenous danger signals (DAMPs) such as uric acid, host DNA, ATP or HMGB1.^2–5^

Squalene-based oil-in-water emulsions such as MF59 and AS03 represent an important class of adjuvants approved for use in human vaccines. The Adjuvant System AS03 contains *α*-tocopherol, squalene and polysorbate-80 and is used in licensed pandemic influenza vaccines. AS03 increases the magnitude and breadth of humoral and cell-mediated immune responses, resulting in improved protection against flu as compared to non-adjuvanted vaccines.^6^ Comparison to alum or AS04 in a phase II randomized trial highlighted the capacity of AS03 to elicit strong antibody responses against a protein antigen.^7^ Mouse and human studies have demonstrated that AS03 triggers transient innate immune responses.^8–10^ The immunostimulatory capacity of AS03 and MF59 relies on their physical and chemical properties.^8,11–14^ Furthermore, the adjuvant properties of MF59 are decreased in absence of the TLR/IL1R adaptor molecule MyD88 or the apoptosis-associated speck-like protein containing CARD (ASC) molecule in an inflammasome-independent fashion.^15,16^ MF59 also induces a local release of extracellular ATP that contributes to the induction of adaptive responses.^4^ Taken together, these data suggest that oil-in-water emulsions trigger cell injury or stress that lead to the release of DAMPs and subsequent innate immune responses. However, the precise mechanisms whereby these clinical adjuvants activate the innate immune system remain largely unresolved.

The objective of this study was to define the early molecular events that underlie the immunostimulatory properties of AS03. We show herein that AS03 induces a rapid perturbation of lipid metabolism in monocytic cells, leading to endoplasmic reticulum (ER) stress and activation of the unfolded protein response pathway. We further unravel how this metabolic perturbation leads to cytokine production by innate cells and contributes to the adjuvant properties of AS03.

## Results

### Upon intramuscular injection, the oil-in-water emulsion AS03 induced profound changes in gene expression in the draining lymph node

**Fig. 1:**
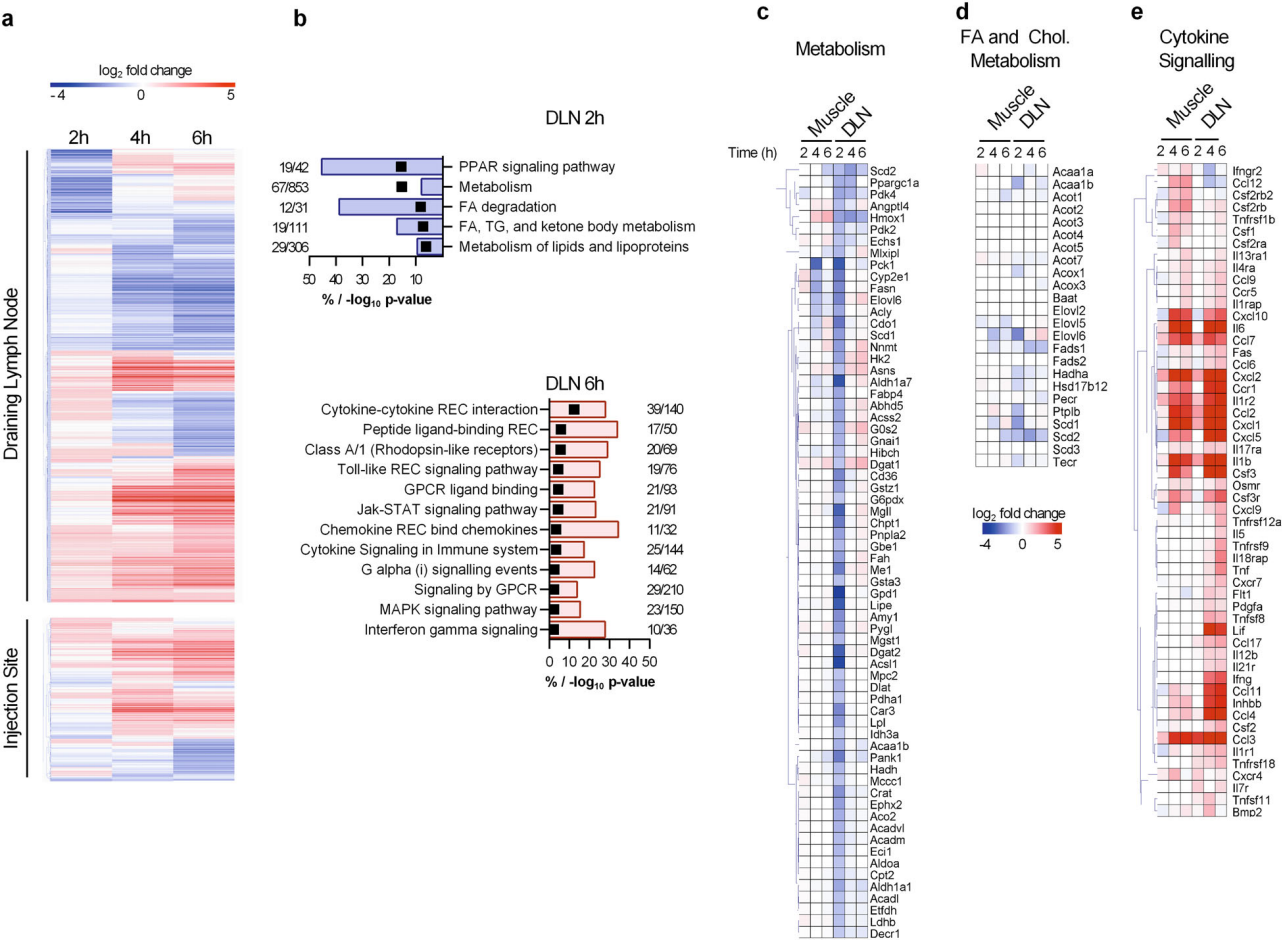
AS03 induces alterations in gene expression in the draining lymph node. Mice received an intramuscular injection of AS03 and the iliac dLN and injection site (muscle) were recovered after 2, 4 or 6 h. **a** Heatmap representation of hierarchically clustered differentially expressed genes (Log2 fold changes vs PBS > 1 or <1 and *p*-value < 0.05) in the dLN or injection site at 2, 4 and 6 h. **b** Overrepresented pathways encompassing significantly downregulated genes (*p*-value < 0.05 and fold change < 0.5) at 2 h and significantly upregulated genes (*p*-value < 0.05 and fold change > 2) at 6 h in the draining lymph node. The bars represent the percentage of down- or upregulated genes and the black boxes represent –log10 (*p*-value). C-E) Heatmap representation of genes belonging to the metabolism (**c**), fatty acid metabolism (**d**) and cytokine-cytokine receptor interaction (**e**) pathways. Each condition represents data obtained from three pools of two mice

To define the early events triggered by AS03, mice were intramuscularly injected with the adjuvant alone or PBS as a control. Gene expression at the injection site (tibialis muscle) and iliac draining LN (dLN) were assessed by microarray analysis. When normalized with respect to PBS, more than two-fold changes (at 2, 4 or 6 h) in gene expression were detected for 1391 probe sets in the dLN and 533 probe sets in the injection site (Fig. 1a). Gene expression changes were therefore more abundant in the dLN than at the injection site. The number of differentially expressed genes increased as time progressed from 2 to 6 h (Fig. 1a). In the muscle at 2 h, changes in gene expression were infrequent (61/533 probe sets), the major cluster of probe sets representing increases in gene expression at 6 h (347/533 probe sets). In the dLN, both increases and decreases in gene expression appeared to be equally represented. In line with previous observations showing that AS03 components are rapidly drained to the LN within 30 minutes,^11^ we observed important changes in gene expression at 2 h in the dLN. One major cluster showed downregulation in gene expression at 2 h with a return to baseline expression at 4 or 6 h (265/1391 probe sets). The other major clusters encompassed genes with baseline expression at 2 h, followed by either increases or decreases in expression (676/1391 probe sets).

Pathway analysis revealed that the early cluster of down-regulated genes at 2 h in the draining lymph node was mostly related to lipid metabolism (peroxisome proliferator-activated receptor γ signalling, metabolism, and fatty acid metabolism pathways) (Fig. 1b). Expression of several enzymes involved in metabolism (Fig. 1c) and more specifically fatty acid metabolism (Fig. 1d), such as the fatty acid elongase ELOVL6 and desaturase SCD1 was transiently downregulated in the dLN at 2 h. At later time points, the upregulated probe sets were enriched in genes belonging to several immune-related pathways both at the injection site and in the dLN (Figs. 1b, e). This could potentially reflect the activation of resident innate immune cells such as macrophages or of neutrophils and monocytes that are recruited to the injection site and dLN.^8^ Major pathways included cytokine-cytokine interaction, MAPK and STAT signalling (Fig. 1b). As previously noted,^8^ increased expression of multiple genes encoding cytokines or chemokines such as CXCL1, CXCL2, CSF3, IFNγ, IL6, IL1β, CCL2, CCL3, CCL4 and CCL7 was observed both at the injection site and in the dLN (Fig. 1e). These in vivo transcriptomic data indicate that AS03 triggered a potent innate immune response in the dLN and to a lesser extent at the injection site. In the dLN, these events were preceded by a transient modulation of the expression of genes involved in lipid metabolism, which was less apparent at the injection site.

### The oil-in-water emulsion AS03 induced profound modifications of gene expression and lipid content of RAW macrophages

**Fig. 2:**
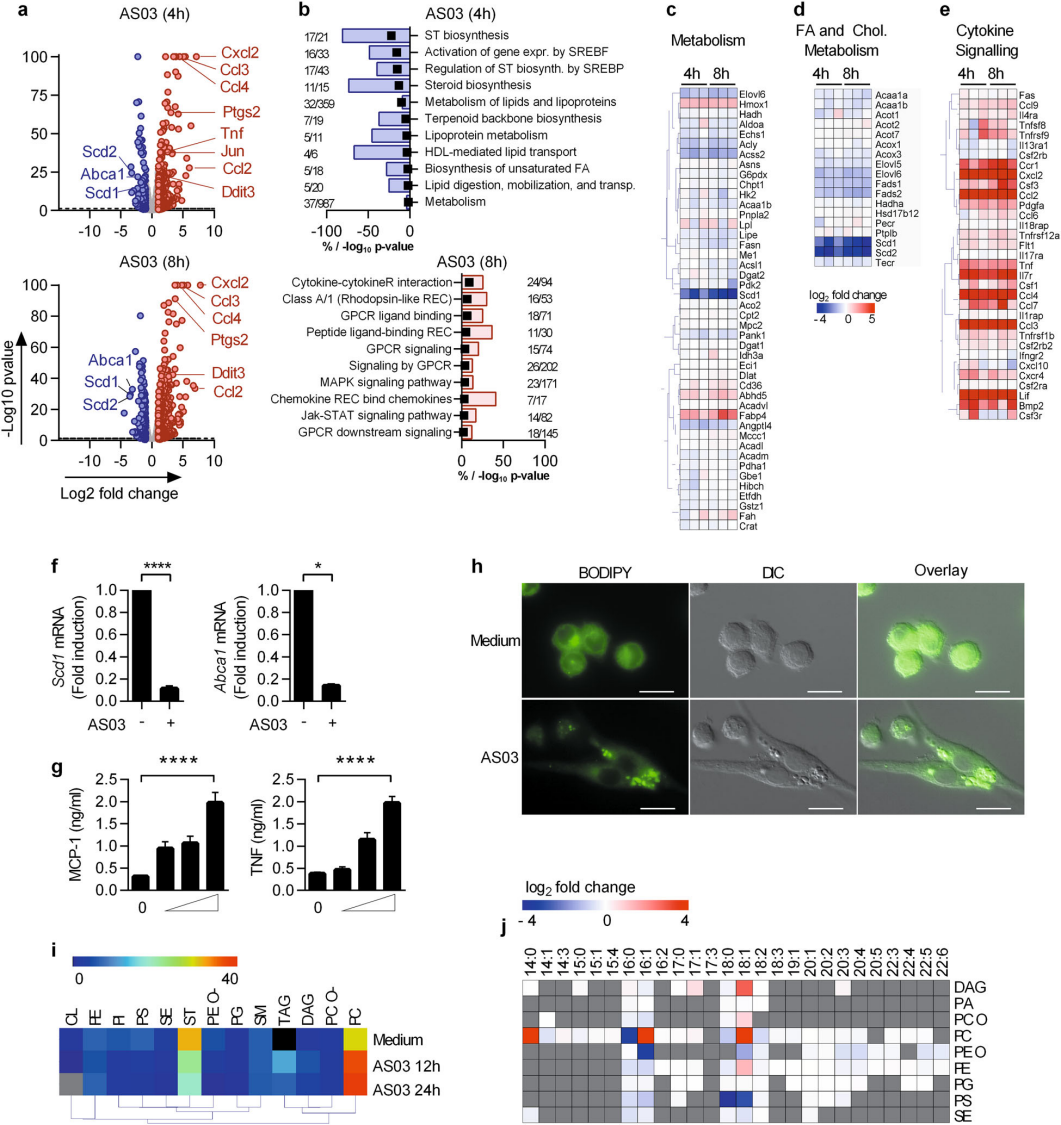
AS03 promotes rapid modifications of gene expression and lipid content in RAW 264.7 macrophages. **a**–**e** RAW 264.7 cells were stimulated with AS03 for 4 or 8 h, and gene expression was assessed by RNA sequencing (**a**) Volcano plot of RNA sequencing data. Each point represents one gene plotted by log2 fold change (FC) vs medium against –log10 of the *p*-value (average of triplicates). The horizontal bar represents a *p*-value of 0.05. The light grey points represent genes with FC < 2 and FC > 0.5, blue points represent FC < 0.5 and red points FC > 2. **b** Overrepresented pathways representing significantly downregulated genes (*p*-value < 0.05 and fold change < 0.5) at 4 h and significantly upregulated genes (*p*-value < 0.05 and fold change > 2) at 8 h. The bars represent the percentage of down- or upregulated genes and the black boxes represent –log10 (*p*-value). **c–e** Heatmap representation of genes previously identified as differentially regulated in vivo, related to the metabolism (**c**), biosynthesis of unsaturated fatty acid (**d**) and cytokine-cytokine receptor interaction pathways (**e**). The colour-coded scale representing fold change vs medium (blue = downregulated vs medium, red = upregulated vs medium) is shown below. **f** mRNA levels for SCD1 and ABCA1 in the RAW cells in response to AS03 (1/30) (normalized with β-actin housekeeping gene, fold change vs unstimulated). *p < 0.05 determined by Mann–Whitney test. **g** MCP-1 and TNF production after stimulation of RAW cells with increasing doses of AS03 (1/500, 1/100, 1/30; mean ± SEM, *n* > 3). *p < 0.05 determined by Mann-Whitney test on medium vs AS03 1/30. The data is representative of at least 6 independent experiments performed in triplicates. **h** Neutral lipid stain (BODIPY 493/503) on RAW 264.7 cells analysed by fluorescence microscopy after 6 h of incubation with medium alone, or AS03 (1/30), scale bar: 20 µm). One representative experiment out of 3. **i** Heatmap depicting the proportion (in percentage of total lipid content) of different lipid classes in RAW cells stimulated 12 or 24 h with medium and AS03. Cardiolipid (CL), phosphatidylethanolamine (PE), phosphatidylinositol (PI), phosphatidylserine (PS), Sterol esters (SE), cholesterol (ST), phosphatidylethanolamine–ether (PE O–), phosphatidylglycerol (PG), sphingomyelin (SM), triacylglycerol (TAG), diacylglycerol (DAG), phosphatidylcholine–ether (PC 0–), phosphatidylcholine (PC). Grey box: below detection level. **j** Heatmap representation of significant (*p* < 0.05) AS03-induced alterations in lipid saturation for each main lipid class (log2 FC AS03 vs medium). The data represents the mean values obtained from three independent samples for each time point

Based on these observations, we hypothesized that metabolic perturbations in lymph node-resident innate immune cells could contribute to the immunostimulatory properties of AS03. Adjuvants consisting of squalene-containing emulsions have been shown to target macrophages in the draining lymph nodes.^17,18^ In order to decipher the molecular events triggered by AS03 and in keeping with the in vivo data, we further studied the effect of AS03 upon in vitro stimulation of macrophages. Transcriptomic analysis revealed that AS03 induced an important modulation (both increases and decreases) of gene expression in RAW 264.7 macrophage cells. We observed a significant downregulation of 109 and 214 genes at 4 h and 8 h time-points, respectively (Fig. 2a). Importantly, as observed following in vivo immunization, pathway enrichment analysis (Fig. 2b) revealed that these downregulated genes were related to metabolism (Fig. 2c) and more specifically to fatty acid and cholesterol metabolism (Fig. 2d). These results were validated by RTqPCR for *Scd1* and *Abca1* (Fig. 2f). 273 and 355 genes were significantly up-regulated at 4 and 8 h time-points, respectively (Fig. 2a). Again, several innate immune-related pathways that were overrepresented in vivo were also identified in this in vitro model (Fig. 2b). In particular, multiple genes encoding chemokines and cytokines such as CXCL2, CCL2, CCL3, CCL4, CCL7 and CSF3 were found among the most differentially regulated genes (Fig. 2e). As previously noted for human monocytes,^8^ we confirmed the capacity of RAW 264.7 cells to produce inflammatory mediators such as TNFα or MCP-1 in response to AS03 (Fig. 2g).

Alterations in the expression of genes involved in lipid metabolism both in vitro and in vivo suggest that total cellular lipid content may be affected by AS03. Staining of neutral lipids with a lipophilic fluorescent dye revealed that AS03 induced the rapid formation of cytoplasmic lipid droplets, organelles that are involved in lipid storage and trafficking (Fig. 2h). In order to more specifically identify lipid alterations in these cells, major lipid species and their fatty acid compositions were quantitatively determined by shotgun lipidomics (Figs. 2i, j, respectively). Stimulation with AS03 induced profound alterations in the proportions of different lipid classes. Stimulated cells displayed decreased cholesterol molar fractions at 24 h as well as increases in triacylglycerol (TAG) and phosphatidylcholine (PC) molar fractions (Fig. 2i). The decrease in cholesterol is consistent with the fact that gene expression of multiple enzymes involved in cholesterol biosynthesis was down-regulated (Fig. 1b). The higher content in PC was linked to increases in mono-unsaturated oleic (C18:1), palmitoleic (C16:1)) and saturated myristic fatty acids (C14:0) (Fig. 2j). Polysorbate 80, one of the three components of AS03, is mainly composed of oleic acid. Exogenous oleic acid is mainly incorporated into cellular PC in RAW cells,^19^ which could explain the increase in C18:1 observed in this lipid class. Furthermore, decreased expression of *Scd1* and *Scd2* in AS03-stimulated RAW cells (Fig. 2d) could be secondary to the increase of C16:1/C16:0 and C18:1/C18:0 ratios observed for PC. Taken together, these data indicate that upon internalization by RAW macrophages, AS03 triggered the production of inflammatory mediators and induced acute modifications in cholesterol and fatty acid cellular content.

### AS03 alters the morphology of the endoplasmic reticulum and triggers an 'ER stress' response in RAW cells

**Fig. 3:**
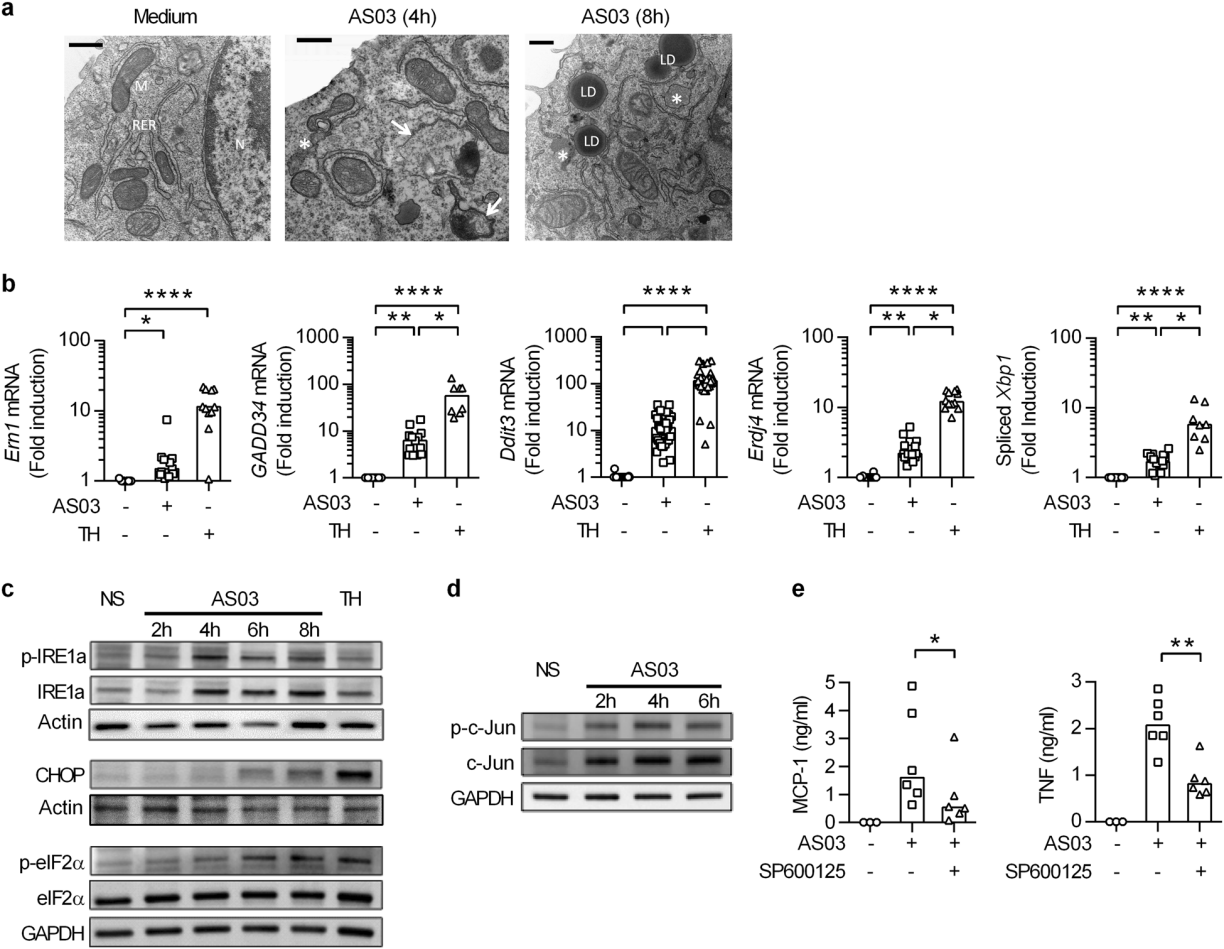
AS03 induces activation of the ER stress pathway. **a** RAW cells were treated as indicated, fixed and processed for TEM. Arrows, mitophagy; asterisks, swollen ER; *M* mitochondrion, *N* nucleus, *LD* lipid droplets. (Scale bars: 500 nm). One representative experiment out of three. **b** Expression of *Ern1* and *Gadd34, Ddit3, Erdj4 and* spliced *Xbp1* in RAW cells in response to AS03 (1/30) and thapsigargin as a positive control (TH; 10 μM) at 6 and 4 h respectively and measured by qPCR (normalized to β-actin and expressed as a fold change vs medium). Statistical significance was determined by a Kruskal-Wallis test followed by Dunn’s multiple comparisons. The data is representative of at least 6 independent experiments performed in triplicates. **c** Immunoblot detection of the ER stress markers p-IRE1α, p-eIF2α and CHOP in RAW cells in response to AS03 (1/30; 2–8 h) or Thapsigargin (TH; 10 μM; 4 h). **d** Detection of phosphorylated c-Jun by Western Blot in RAW cells in response to AS03 (1/30; 2–6 h). The data is representative of three independent experiments (**e**) ELISA detection of MCP-1 and TNF-α production by RAW cells 24 h after AS03 stimulation (1/30) with or without the JNK inhibitor SP600125 (10 μM, *n* = 6 independent experiments). Statistical significance was determined by Wilcoxon paired test

The ER is the major site of lipid biogenesis. Alteration of phospholipid composition and consequent changes in membrane rigidity and fluidity have been shown to jeopardize ER homeostasis, a situation referred to as ER stress.^20^ In electron micrographs of untreated RAW cells, the ER appeared as normal tubular cisternae (Fig. 3a). In sharp contrast, cells treated for 4 h and 8 h with AS03 contained numerous dilated structures delimited by electron-dense ribosomes (Fig. 3a). As observed in the context of lipotoxic cellular stress,^21^ these results indicate that AS03 strongly altered ER morphology. Perturbation of ER homeostasis activates a set of intracellular signalling pathways known as the unfolded protein response (UPR). We observed that AS03 induced the upregulation of classical UPR signature genes such as *Ern1, Gadd34, Ddit3* (encoding C/EBP homologous protein (CHOP), *Erdj4* and spliced *Xbp1* (Fig. 3b). Thapsigargin, an inhibitor of the Sarcoplasmic/endoplasmic reticulum calcium ATPase was used in parallel as a positive control. The most conserved ER-anchored stress sensor of the UPR consists of inositol requiring enzyme 1α (IRE1α, encoded by *Ern1*). Its cytosolic domain contains dual catalytic functions of an autophosphorylating serine/threonine kinase and an endoribonuclease (RNase). We observed phosphorylation and upregulation of IRE1α upon AS03 treatment along with CHOP induction and phosphorylation of eIF2α, indicative of ER stress (Fig. 3c). Through the recruitment of TRAF2, the cytoplasmic domain of IRE1α may lead to activation of c-Jun amino-terminal kinases (JNK).^22^ AS03 induced progressive c-Jun phosphorylation (on serine 63) and accumulation (Fig. 3d). Pharmacological inhibition of JNK by SP600125 decreased the capacity of AS03 to elicit MCP-1 and TNFα production by RAW cells, indicating that this pathway is important for downstream inflammatory gene activation (Fig. 3e). Taken together; these results indicate that the oil-in-water emulsion AS03 alters ER homeostasis in target cells. This was found to be associated with the activation of UPR and JNK-dependent inflammatory pathways.

### IRE1α and ASK1 are required for AS03-induced cytokine production by RAW cells

**Fig. 4:**
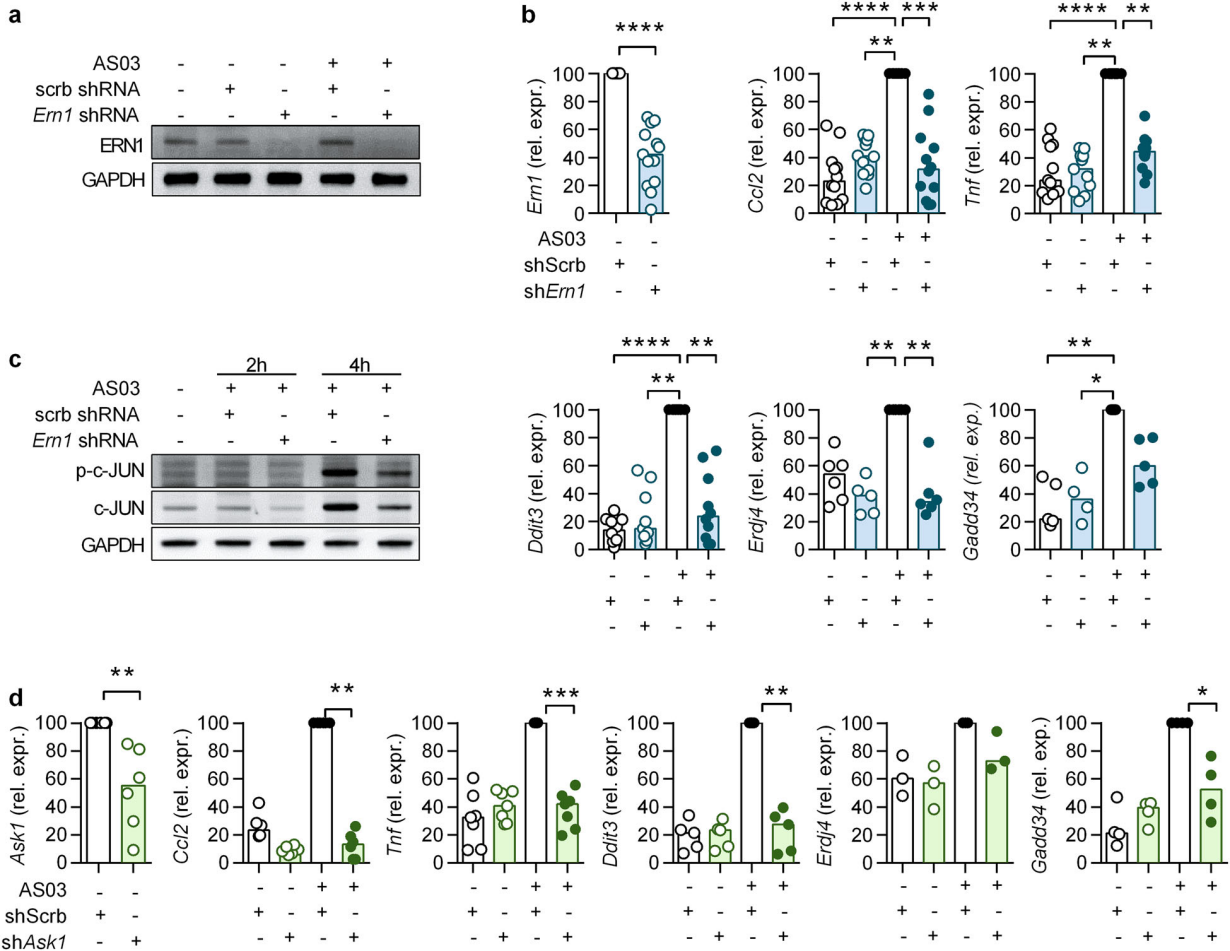
IRE1α and ASK1 are required for cytokine production and ER stress activation for the response to AS03 in RAW cells. **a** Immunoblot detection of IRE1α/Ern1 after AS03 stimulation and IRE1α/*Ern1* knockdown. Representative of two independent experiments. **b** Expression of *Ern1*, *Mcp1*, *Tnf* and the ER stress markers *Ddit3*/*Chop*, *Erdj4* and *Gadd34* mRNAs in RAW cells in response to AS03 (1/30, 6 h) after IRE1α/*Ern1* knockdown. Statistical significance was determined by either a Mann–Whitney test or by a Kruskal-Wallis followed by Dunn’s multiple comparison test. **c** Immunoblot detection of total and phosphorylated c-Jun (S63) after AS03 stimulation and IRE1α/*Ern1* knockdown Representative of two independent experiments. **d** Expression of *Ask1*, *Mcp1*, *Tnf* and the ER stress markers *Ddit3*/*Chop*, *Erdj4*, and *Gadd34* mRNAs in RAW cells in response to AS03 (1/30, 6 h) after *Ask1* knockdown. *p < 0.05 determined by Mann–Whitney. Each dot represents the mean value of an individual experiment performed in triplicates

In order to further define the molecular links between UPR and activation of inflammatory genes by AS03, we performed shRNA experiments targeting *Ern1* in RAW cells. Using this strategy, we obtained efficient knock-down of IRE1α at the mRNA and protein levels (Figs. 4a, b). Transduction with *Ern1* shRNA decreased the capacity of AS03 to induce the expression of inflammatory genes such as *Ccl2* and *Tnf* as compared to scramble shRNA conditions. It also reduced expression of the ER stress-related genes *Ddit3*, *Erdj4*, and *Gadd34* (Fig. 4b). We observed that AS03-induced c-Jun phosphorylation and accumulation were affected by IRE1α knock-down (Fig. 4c). Using the same strategy, we investigated the role of apoptosis signal-regulating kinase 1 (ASK1) (Fig. 4d), a direct target of the IRE1α–TRAF2 complex responsible for ER-stress induced JNK phosphorylation.^23^ Knock-down of ASK1 decreased AS03-induced *Ccl2* and *Tnf* expression along with *Ddit3* and *Gadd34* (Fig. 4d). Taken together, these results indicate that activation of inflammatory genes by AS03 is dependent on the IRE1α/ASK1/JNK pathway.

### 4-PBA treatment hampers the adjuvant properties of AS03

**Fig. 5:**
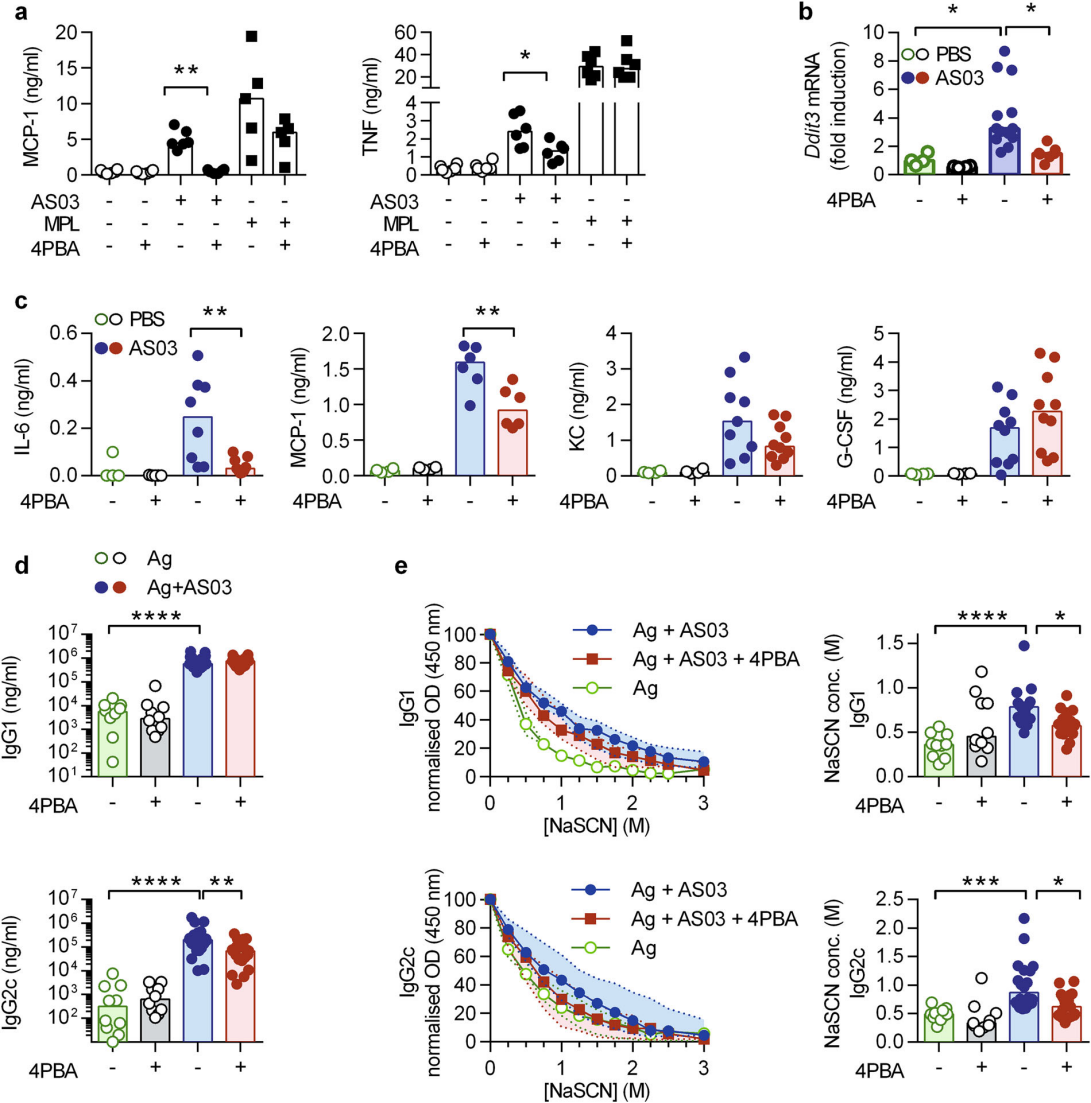
4-PBA inhibition of ER-stress in vivo affects antibody avidity. **a** MCP-1 and TNF secretion 24 h after stimulation with AS03 (1/30) or MPL (10 μg/ml), with 4PBA (3 mM) treatment 1 h prior stimulation. *p < 0.05 determined by Mann–Whitney test on AS03 1/30 vs AS03 + 4PBA. Each dot represents the mean value of independent experiments performed in triplicates and the horizontal bars represent the medians. **b** Expression of *Ddit3/Chop* mRNA in peritoneal cells 6 h after AS03 i.p. injection. **c** Serum cytokine levels 6 h after i.p. injection of AS03, following 3 days of 4PBA-IP injection (2 mg/mouse). Each point represent a single mouse and the horizontal bar represents the median. *p < 0.05 determined by Mann–Whitney. The data is representative of at least two independent experiments. **d** Serum anti-HBs IgG1 and IgG2c titres were measured by ELISA on day 21 (7 days post second immunization). Statistical significance was determined by Kruskal–Wallis followed by Dunn’s multiple comparison test. Each dot represents a single mouse and the horizontal bar represents the median. Data were obtained from two independent experiments. **e** The avidity of the HBs-specific IgG1 and IgG2c antibodies was measured by ELISA with serial dilutions of a chaotropic agent (NaSCN), and represented as both the median and interquartile range of normalized optical density values (0 M NaSCN = 100%) and the concentration of NaSCN required to dissociate 50% of bound antibodies. Statistical significance was determined by Kruskal–Wallis followed by Dunn’s multiple comparison test

The chemical chaperone, 4-phenyl butyric acid (4-PBA) can alleviate ER stress^24^ and therefore provides an experimental opportunity to approach the role of the UPR pathway in the immunostimulatory properties of AS03. In vitro, pre-treatment of RAW cells with 4-PBA strongly decreased AS03-induced MCP-1 and TNFα production. In contrast, it did not affect the capacity of RAW cells to produce these inflammatory mediators in response to the TLR4 ligand MPL (Fig. 5a).

Next, we assessed the effect of 4-PBA on in vivo responses to AS03. For this purpose, we administered both AS03 and 4-PBA intraperitoneally. In control mice, AS03 induced an increase in the expression of *Ddit3* in peritoneal cells, a classical marker of ER stress. Pre-treatment with 4-PBA strongly reduced the induction of *Ddit3* (Fig. 5b). In line with this, IP injection of AS03 increased circulating levels of cytokines and chemokines such as IL-6, MCP-1, KC or G-CSF. Upon 4-PBA treatment, we observed a significant decrease in IL-6 and MCP-1 levels. In contrast, KC and G-CSF levels were comparable in these two experimental groups (Fig. 5c). These results suggest that ER stress might contribute to the immunostimulatory properties of AS03 in vivo. To identify a possible contribution of ER stress in the adjuvant properties of AS03, we modified the experimental set-up. Mice were given 4-PBA per os 3 days prior to vaccination and kept on 4-PBA throughout the experiment. Control or 4-PBA-treated mice were then immunized intramuscularly with either HBsAg or HBsAg adjuvanted with AS03 following a prime (at day 0)-boost (at day 14) regimen, and the Ag-specific antibody levels were measured at day 21. HBsAg is a relevant antigen for human vaccines as it is used in the *Engerix* B vaccine. While AS03 induced comparable levels of HBsAg-specific IgG1 antibody titres in control and 4-PBA-treated mice, IgG2c titres were significantly decreased in the 4-PBA treated group (Fig. 5d). In order to evaluate the quality of the antibody response, we also analysed the avidity of IgG antibodies using a classical chaotrope-based assay.^25^ Increased antibody avidity reflects affinity maturation of B cells in the germinal center via somatic hypermutation.^26^ In control mice, AS03 increased the avidity of HBsAg-specific antibodies as compared to mice immunized with antigen alone. Importantly, these avidity indices were decreased in 4-PBA-treated mice for both HBsAg-specific IgG1 and IgG2c antibodies (Fig. 5e). These results suggest that ER stress-related pathway does likely contribute to the adjuvant properties of AS03 in vivo.

### *IRE1α* expression by myeloid cells is involved in the adjuvant effect of AS03

**Fig. 6:**
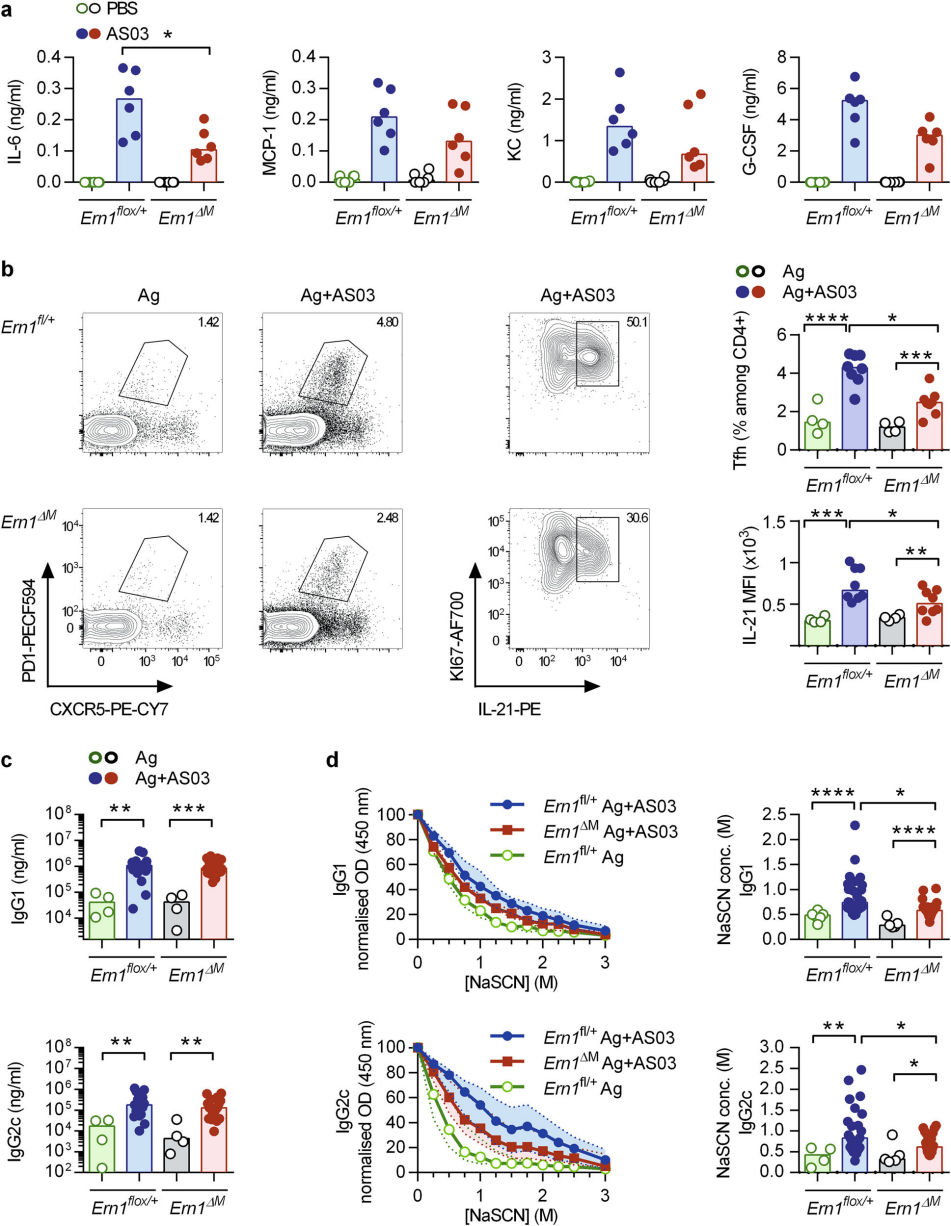
*IRE1α* expression by myeloid cells is required for optimal response to AS03 adjuvant. **a** Ern1^flox/+^ and Ern1^ΔM^ mice were injected i.m. with PBS or AS03. 6 h later, serum was collected and cytokine (IL-6, MCP-1, KC, G-GSF) production was measured by ELISA. Each point represents a single mice and the horizontal bar represents the median. *p < 0.05 determined by Mann–Whitney. Data is representative of two independent experiments. **b** Ern1^flox/+^ and Ern1^ΔM^ mice were immunized with HBsAg or HBsAg + AS03 and the DLNs were recovered on day 7 and analysed for Tfh markers or stimulated with PMA/ionomycin in the presence of brefeldin A and stained for Ki67 and IL-21 expression (**c**) Ern1^flox/+^ and Ern1^ΔM^ mice were immunized with HBsAg or HBsAg + AS03 (d0) and a boost was performed on day 14. Serum anti-HBs IgG1 and IgG2c titres were measured by ELISA on day 21. Statistical significance was determined by Kruskal-Wallis followed by Dunn’s multiple comparison test. Each dot represents a single mouse and the horizontal bar represents the median. Data were obtained from two independent experiments. **d** The avidity of the HBs-IgG1 and IgG2c was measured by ELISA with serial dilutions of a chaotropic agent (NaSCN) and represented as both the median and interquartile range of normalized optical density values (0 M NaSCN = 100%) and the concentration of NaSCN required to dissociate 50% of bound antibodies. Statistical significance was determined by Kruskal–Wallis followed by Dunn’s multiple comparison test

4-PBA has multiple different targets.^27^ Furthermore, UPR-induced splicing of *Xbp1* is required for differentiation of B cells into plasma cells and therefore for optimal IgG secretion.^28^ Hence, to more specifically address the role of ER stress-related pathway in cells that are directly targeted by AS03, we generated *Ern1*^flox/flox^ LysM-Cre mice (*Ern1*^*ΔM*^) that lack IRE1α in myeloid cells, i.e. macrophages, monocytes and neutrophils. *Ern1*^*ΔM*^ and *Ern1*^*flox/+*^ control mice were injected with AS03 i.m. and cytokine levels were determined in the sera. We observed a significant decrease in IL-6 levels and a trend for lower production of MCP-1, KC and G-CSF (Fig. 6a). Next, we immunized these experimental groups with HBsAg alone or HBsAg formulated with AS03. Optimal B-cell responses require help from T follicular helper cells (T_fh_). These cells are essential for the development of germinal centres and provide help for B-cell affinity maturation and for the development of long-lasting memory B and plasma cells.^29^ As IL-6 is required for efficient differentiation of naïve CD4 T cells into Tfh cells in vivo,^30^ we evaluated the proportion of PD1^+^CXCR5^+^ CD4 T cells in the dLN 7 days after immunization. We observed that AS03 promoted a potent T_fh_ response (Fig. 6b). An important proportion of these cells was found to be proliferating (Ki67^+^) and expressing IL-21. In *Ern1*^*ΔM*^ mice, the proportion of T_fh_ cells and their capacity to produce IL-21 was reduced as compared to their littermates. Consistent with the role of T_fh_ cells on antibody affinity maturation, we observed decreased avidity indices in *Ern1*^*ΔM*^ mice despite comparable HBsAg-specific IgG1 and IgG2c titres (Figs. 6c, d). These results indicate that expression of the ER stress sensor IRE1α by myeloid cells is not mandatory for the induction of high Ag-specific antibody titres by AS03. However, this pathway was found to be selectively required for the induction of IL-6 and robust T_fh_ responses, thereby promoting Ag-specific antibody affinity maturation.

## Discussion

Oil-in-water emulsions are now used in several licensed or candidate vaccines. Herein we identify a signaling pathway involved in the adjuvant effect for AS03. This emulsion is rapidly drained to the lymph node where we observed profound modifications in gene expression linked to lipid metabolism. In vitro, AS03 is engulfed by endocytic cells such as macrophages. It altered their cellular lipid content and ER homeostasis, and therefore promoted the activation of the UPR pathway. Blockade of ER stress or of downstream signalling events inhibited both innate and adaptive responses to the adjuvant, decreasing AS03-induced innate cytokine production and the quality of the response against antigens formulated in AS03.

In vivo transcriptomic data revealed distinct kinetics of gene expression profile between the injection site and draining lymph nodes. In the dLN, the response to AS03 was detected earlier (as fast as 2 h), probably reflecting modifications in gene expression in immune cells such as lymph node resident subcapsular sinus macrophages. Indeed, radiolabelled AS03 can be detected in the dLN as soon as 30 minutes following intramuscular injection^11^ and other oil-in-water emulsions rapidly accumulate in the subcapsular region of the dLN.^17,18^ The observed delay in the muscle response (relatively low changes observe at 2 h) and the increase in immune gene expression observed at 4 and 6 h may rather reflect the response of innate cells recruited to the muscle. Indeed, a rapid influx of neutrophils and monocytes in the muscle is observed upon I.M. immunization with MF59.^31^

The first changes in gene transcription detected in the dLN included downregulation of genes linked to lipid metabolism. These observations could be mirrored in vitro in the macrophage cell line RAW264.7. In addition, AS03 induced lipid droplet formation and alterations in the total lipid composition of these cells. Other squalene containing emulsions also induce accumulation of neutral lipids, lipid droplet formation and lipid alterations.^13^ Unfortunately, due to technical limitations, we were not able to assess squalene levels in our shotgun lipidomic analysis. Squalene is converted into free cholesterol and cholesteryl esters leading to a decrease in endogenous cholesterol biosynthesis.^13^ However, AS03 induced a decrease in the relative proportions of both free cholesterol and cholesterol esters. This could be due to either a decrease in total cholesterol content, an enrichment in phosphatidylcholines (PC) or a combination of both events. Transcriptomic analyses revealed a strong decrease in expression of genes involved in de novo cholesterol biosynthesis after AS03 stimulation, indicating a possible negative feedback loop due to the addition of squalene. Conversely, increased PC synthesis could be a response to counteract the possible toxic effects of excess cholesterol in macrophages. Indeed, cholesterol-loaded macrophages increase PC biosynthesis allowing both the sequestration of cholesterol esters in lipid droplets and ER membrane biogenesis.^32^ In addition to squalene, other components of AS03 may be involved in the alteration of lipid metabolism and this would require further investigations. α-Tocopherol represents an important component of AS03 but its exact role remains poorly understood. It protects polyunsaturated fatty acid from peroxidation^33^ and displays multiple cellular functions that are independent of its radical scavenging ability,^34^ including repression of de novo cholesterol biosynthesis by targeting post-transcriptional processing of SREBP-2.^35^ It will be important to define if AS03-derived α-tocopherol accumulates in the cell and its contribution to lipid metabolism.

General alterations in cellular lipid homeostasis can lead to ER stress and the activation of the UPR pathway.^20^ More specifically, ER membrane lipid alterations induce the activation of the transmembrane protein IRE1α.^36,37^ The UPR is activated to maintain ER function and ultimately cell survival, and is likely what occurs in macrophages stimulated with AS03. This pathway plays a central role in immunity in both physiological and pathological settings.^38^ Furthermore, it represents a central hub for the integration of metabolic stress and subsequent initiation of inflammatory responses through multiple mechanisms.^39^ Herein, we show that the IRE1α-TRAF2-ASK1 pathway, leading to JNK and c-Jun activation^22,23^ plays a central role for the induction of cytokine production by AS03. We focused on IRE1α as it intersects with inflammatory pathways.^38^ However, the other two branches of the UPR (PERK/eIF2α and ATF6) could also contribute to this process. Of note, we observed phosphorylation of eIF2α and increased expression of CHOP (*Ddit3*) and *Gadd34* which were shown to synergize with TLR signalling pathways.^40,41^ The potential role of these pathways in the adjuvant properties of AS03 should be investigated more in-depth.

Our results clearly indicate that multiple pathways are involved in the adjuvant effects of AS03. As shown for MF59, the in vivo release of DAMPs such as ATP could be sufficient to elicit local inflammatory response and high Ag-specific antibody titres.^4^ However, other critical parameters of the response to AS03, i.e. robust T_fh_ cell differentiation and affinity maturation were found to be dependent on the UPR in monocytes or macrophages. Lack of IRE1α/*Ern1* expression in these cells resulted in lower T_fh_ cell frequencies and also in decreased T_fh_-derived IL-21, a cytokine with a well-known role in the generation of plasma cells and high affinity antibodies.^42^ We have observed that lack of IRE1α/*Ern1* in monocytes/macrophages or 4-PBA treatment resulted in decreased IL-6 expression. This cytokine plays a major role in the differentiation of T_fh_ cells, but also in T_fh_-derived IL-21 synthesis and in antibody production.^42–45^ Along this line, systems vaccinology approaches revealed that in human subjects vaccinated against influenza virus, early expression of UPR-related molecules were robust biomarker of the later emergence of protective antibody titres.^46^

In conclusion, our results identify altered lipid metabolism and the UPR as important components of the response to an oil-in-water adjuvant. Activation of an inflammatory response following metabolic perturbation may represent an evolutionary conserved mechanism by which cells infected by intracellular pathogens initiate a defensive response^47^ and a valuable target for immunointervention. Furthermore, in the context of vaccination, integration of metabolic stress represent a critical step for the induction of protective immune responses.^48^ Hence, a better understanding of the underlying cellular and molecular mechanisms may help improve current adjuvant formulations.

## Materials and methods

### Cells and reagents

RAW 264.7 (ATCC) cells were cultured in medium (DMEM containing 10% of FCS, L-glutamine, pyruvate, non-essential amino acids, and penicillin/streptomycin/gentamycin) in CellStar petri dishes (Greiner Bio One) at 37°C with 5% of CO_2_. AS03 (Adjuvant System 03) with or without Hepatitis B surface antigen (HBsAg) were provided by GSK. AS03 used in these experiments is composed of 23,72 mg/ml α-tocopherol, 21,38 mg/ml squalene and 9,72 mg/ml polysorbate-80 in PBS. The AS03 was used at the final concentration (Vol/vol) of 1/30 when used in vitro unless stated otherwise. The following reagents were used at the indicated concentrations or following manufacturer’s indications: SP600125 (10 μM - Tocris Bioscience), Monophosphoryl lipid A (MPL, 10 μg/ml resuspended in distilled water—GSK), Thapsigargin (TH, 10 μM—Sigma).

### Mice

C57BL/6 mice (6 weeks old) were purchased from Envigo. Myeloid-specific ERN1-deficient mice (*Ern1*^ΔM^) were generated by crossing *Ern1*^flox/flox^ mice^49^ (B6;129S4-Ern1 < tm2.1Tiw>, obtained from Riken BRC, Japan and further backcrossed on C57BL/6 background for 5 generations) with LysM-Cre mice (B6.129P2-Lyz2tm1(cre)Ifo/J, Jackson lab). Mice used in this study were housed and bred under specific pathogen-free conditions. The experiments were carried out according to the laws and regulations of the institution and were approved by the local ethics committee of the Biopole ULB Charleroi (BUC).

### In vivo studies

The chemical chaperone 4-PBA (2 mg/mouse, resuspended in physiological serum (Bioconnect)) was administered to mice one hour before injection of 50 µl AS03 containing 1 μg of HBsAg and 2 µl of AS03 intraperitoneally. For adaptive studies, 4-PBA was resuspended in distilled water, filtered and further diluted in the drinking water at a concentration of 1 g/kg/day. Three days after, mice were immunized with 20 μl of a formulation containing HBsAg (1 μg) in PBS or in physiological serum containing 2 μl of AS03 in each gastrocnemius muscle of both hind limbs.

### Microscopy

For neutral BODIPY staining, 2 × 10^5^ RAW cells were seeded in each compartment of compartmented 35-mm sterile culture dishes with glass bottom (Cellview – Greiner). Cells were incubated for 4 h with AS03. Coverslips were washed and cells were stained with BODIPY 493/503 (Thermofisher) (1/500) and visualized with a Zeiss Axio Observer wide-field microscope.

For TEM, samples were washed with PBS and fixed with ice-cold glutaraldehyde 2% (EM grade, Sigma). Then, they were post-fixed in OsO_4_ (2%) in 0.1 M cacodylate buffer (pH 7.2), serially dehydrated in increasing ethanol concentrations, embedded in Agar 100 resin (Agar Scientific Ltd, UK) and left to polymerize at 60°C for 2 days. Ultrathin sections (50–70 nm thick) were produced with a Leica EM UC6 ultra-microtome, collected on formvar-carbon-coated copper grids and stained with uranyl acetate and lead citrate by standard procedures. Observations were made on a Tecnai 10 TEM (FEI) and images were captured with a Veleta CCD camera and processed with SIS iTEM (Olympus).

### In vivo transcriptomic analysis

Mice were injected in each gastrocnemius muscle of both hind limbs with AS03 or PBS for 2, 4 and 6 h. The draining lymph node and the injection site were collected (3 pools from 2 mice for each sample), washed with PBS and lysed in 1 ml Tripure reagent (Roche Applied Science). RNA was extracted with chloroform and purified with the RNeasy Minikit (Qiagen) following manufacturer’s instructions, with DNase treatment of the column to remove possible genomic DNA contamination. RNA was concentrated by ethanol precipitation and quantified by RiboGreen (Life Technologies). One μg of each RNA sample was used for target preparation, using a one-cycle cDNA synthesis kit and hybridized to GeneChip Whole Mouse Genome 430 2.0 arrays (Affymetrix). Data acquisition was performed using GeneChip Operating Software (Affymetrix) and data quality control and normalisation was performed with the R and Bioconductor stats packages. Fold changes (AS03 *vs* PBS) were calculated for each sample and a heatmap of differentially expressed genes (FC < 0.5 or >2, *p*-value < 0.05) (were generated with MeV software or GraphPad Prism software. Pathway over-representation of significantly upregulated genes (*p*-value < 0.05 and fold change > 2) was performed with the InnateDB web resource using the hypergeometric algorithm and Benjamini-Hochberg correction for *p*-values.^50^

### In vitro transcriptomic analysis

10^6^ RAW 264.7 cells were stimulated with AS03 (1/30) for 4 or 8 h in independent triplicates, and total RNA was extracted with an RNeasy Plus Mini Kit (QIAGEN) following manufacturer’s instructions. The RNA concentration and purity were measured with a NanoDrop spectrophotometer (A260/280 and A260/230 > 1.8). About 400 ng of total RNA were used for TruSeq Library preparation (Illumina) according to the manufacturer’s recommendations and the sequencing was carried out on the NextSeq platform (Illumina). The sequencing and analysis were performed by VIB Nucleomics Core (KULeuven, Belgium). A volcano plot was generated with the average fold change and p-values for each gene using GraphPad Prism software. Pathway over-representation of significantly upregulated genes (*p*-value < 0.05 and fold change > 2) was performed with the InnateDB web resource using the hypergeometric algorithm and Benjamini-Hochberg correction for p-values.^50^ A heatmap of genes previously identified in vivo belonging to the metabolism, biosynthesis of unsaturated fatty acid and cholesterol metabolism pathways (FC > 2 or <0.5) was generated with MeV software.

### Quantitative profiling fatty acid composition and of lipids

Lipid analysis was performed by Lipotype (Dresden, Germany). Lipids were extracted using two-step lipid extraction using Chloroform and Methanol.^51^ Samples (60,000 cells) were spiked with lipid class-specific internal standards prior to extraction. Lipid extracts were immediately subjected to mass spectrometric analysis. Mass spectra were acquired on a hybrid quadrupole/Orbitrap mass spectrometer (Q-Exactive, Thermo-Fisher) equipped with an automated nano-flow electrospray ion source (Triversa Nanomate, Advion) in both positive and negative ion mode. Lipid identification using LipotypeXplorer^52^ was performed on unprocessed mass spectra. In case of MS-only experiments, lipid identification is based on the molecular masses of the intact molecules. In case of MS-MS experiments including the collision-induced fragmentation of lipid molecules, lipid identification is based on both the intact masses and the masses of the fragments. Lipid identifications were filtered according to spectral noise, background signals, mass accuracy and occupation threshold (i.e., only lipids identified in all replicate samples were considered for quantification) prior to normalization and further statistical analysis. Lists of identified lipids and their intensities were stored in databases optimized for the particular structure inherent to lipidomic datasets. Lipid class-specific internal standards were used for lipid quantification. For data handling, normalization and graphical visualization, modules and tools implemented into the Lipotype LIMS were used.

### Quantitative real time PCR

mRNA content of 3 × 10^5^ RAW cells was isolated with the MagNA Pure LC mRNA isolation kit (Roche) on the MagNA pure instrument (Roche Applied Science) following manufacturer’s indications. *Tnf, Il6, Mcp1, Scd1, Anca1, Ern1, Gadd34, Ddit3/Chop, Erdj4, Ask1, Actin*, and *Gadph* mRNA levels were quantified by real-time PCR using TaqMan RNA Amplification Kit (Roche) or LightCycler Multiplex RNA Virus Master (Roche) on a LightCycler 480 instrument (Roche Applied Science). Primers and probes were synthesized by Eurogentec (*Tnf*: F: cagaccctcacactcagatca, R: cacttggtggtttgctacga, probe tcgagtgacaagcctgtagccca, *Il6*: F: gaggataccactcccaacagacc, R: aagtgcatcatcgttgttcataca, probe cagaattgccattgcacaactcttttctca, *Mcp1*: F: cttctgggcctgctgttca, R: ccagcctactcattgggatca, probe ctcagccagatgcagttaacgcccc, *Scd1*: F: ttcttctctcacgtgggttg, R: cgggcttgtagtacctcctc, probe cgcaaacacccggctgtcaa, *Abca1*: F: ggaagggtttctttgctcag, R: caaagggtggcacaatcag, probe ccagctgtctttgtttgcattgccc, *Ern1*: F: acgaaggcctgacgaaact,R: tttacccatgtagaggattcca, probe accatcccagaattggttcaggcc, *Gadd34*: F: gcttttggcaaccagaacc, R: gactgagcaagcccatcagt, probe cgcccacaacttctatctcctgtcc, *Ddit3*/*Chop*: F: ccacacctgaaagcagaacc, R: accgtctccaaggtgaaagg, probe ctgccatgactgcacgtggacc, *Erdj4*: F: Tgctgaagcaaaattcagaga, R: tccaattgtgtcatactctttcc, probe ccgagagtgtttcatacgcttctgca, *Ask1*: F: ctcaagtcccagcccataga, R: tcagaatcttccgtggtcgt, probe ccctgggtttcctgtgtgcca, *Actb*: F: agtccgcagcactcagactatgtgca, R: agtccgcagcactcagactatgtgca, probe atcggtggctccatcctggc, *Gapdh*: F: attgtcagcaatgcatcctg, R: ccttccacaatgccaaagtt, probe ccctggccaaggtcatccatga). mRNA levels were normalized to *Gapdh* mRNA expression.

### SDS-PAGE and western blotting

1.5 × 10^6^ RAW cells were rinsed with PBS and lysed in 60 µl RIPA buffer (PBS with 1% Igepal CA-630, 0.5% Na deoxycholate and 0.1% SDS) containing protease (cOmplete Mini Protease Inhibitor Cocktail Tablet, Roche) and phosphatase inhibitors (PhosSTOP, Roche). Lysed cells were incubated on ice for 20 min, cleared by centrifugation at 12,000*g* for 20 min at 4 °C and stored at −80 °C. Protein concentration was measured with the Micro BCA Protein Assay kit (Pierce) and 20 µg of protein was loaded onto a 12% Bis-Tris polyacrylamide gel. Gels were run in NuPAGE MOPS SDS Running Buffer (Invitrogen) at 150 V for 1 h. Proteins were transferred onto a PVDF membrane (Amersham) for 1 h at 100 V, blocked in TBS-Tween containing 5% BSA and blotted with rabbit anti-phospho-IRE1α (S724) (Abcam, ab48187 1/1000), rabbit anti-phospho-eIF2α (S51) (Cell Signaling #9721 1/1000), rabbit anti-phospho-JNK (Thr183/Tyr185) (Cell Signaling #9251 1/1000), rabbit anti-phospho-c Jun (S63)(Cell Signaling, #9261 1/1000), rabbit anti-IRE1α (Cell Signaling, #3294, 1/1000), rabbit anti-eIF2α (Cell Signaling, #9722, 1/1000), rabbit anti-JNK (Cell Signaling, #9252 1/1000), rabbit anti-c Jun (Santa Cruz, sc-1694, 1/1000), anti-CHOP (Santa Cruz, sc-575, 1/1000), mouse anti-actin (Sigma-Aldrich clone AC-74 – 1/2000) antibodies, mice anti-GAPDH (Meridian Life Science, H86504M, 1/6000) followed by detection with donkey anti-rabbit/mouse IgG-HRP (GE Healthcare – 1/5000). All blots derive from the same experiment and were processed in parallel. Uncropped immunoblots with molecular weights are shown in the supplementary file.

### Enzyme-linked immunosorbent assay (ELISA)

For cytokine measurements, 5 × 10^5^ RAW cells were seeded in 96-well plates, stimulated with AS03 and the supernatants were collected after 24 h. ELISAs (mMCP-1, mTNF) were carried out following manufacturer’s instructions (R&D Systems). For Ag-specific antibody measurements, plates were coated with the HBs protein as the coating antigen and with goat anti-mouse IgG (GAM – SouthernBiotech or Jackson ImmunoResearch) as the coating antibody for the standard curve. Plates were blocked at room temperature for 1 h with PBS-1% BSA. Serial dilutions of serum samples and the IgG standard (SouthernBiotech) were added and incubated for 2 h at room temperature, followed by addition of biotin-conjugated anti-mouse IgG (Jackson Research or SouthernBiotech) and streptavidin-HRP. TMB was used as a substrate and plates were read at 450 nm on a microplate reader. For chaotropic Elisa, the avidity of the HBs-IgG1 and IgG2c were measured by ELISA following the protocol as above, but only one sample concentration was selected and serial dilutions of NaSCN between 2 M and 0 M were for 15 minutes for each sample.

### shRNA generation and lentivirus production

Targeting sequences for mouse *Ask1* (GGAAGGATGAAGATTGAAACT) was identified with the siRNA Wizard v3.1 (InvivoGen). The *Ern1/Ire1a* (GCTCGTGAATTGATAGAGAA) target sequence was previously described in.^53^ The targeting sequences were cloned into the pRSI12-U6-(sh)-HTS4-UbiC-TagRFP-2A-Puro (Cellecta) plasmid according to manufacturer’s instructions and verified by sequencing. These vectors were transiently transfected into HEK293T cells along with the packaging vector psPAX2 (Addgene plasmid 12259) and the VSV-G encoding plasmid pMD2.G as previously described.^54^ The supernatant was harvested 48 h after transfection and viruses were concentrated by ultracentrifugation and resuspended in Opti-MEM medium (Thermo Fisher).

### Flow cytometry

*Ern1*^flox/+^ and *Ern1*^ΔM^ mice were immunized by injection in the gastrocnemius muscle and the iliac lymph nodes were recovered on day 7. Cells were recovered from the lymph nodes in a 12-well plate by grinding in a circular motion with the plunger of a syringe, and were passed through a strainer to eliminate debris. Cells were stained following a standard procedure with FcR blocking reagent (Miltenyi Biotec), Live/Dead Aqua (ThermoFisher Scientific), anti-CD4 (Pacific Blue, clone RM4-5, BD Biosciences), anti-PD1 (PE-CF594, clone J43, BD Biosciences) and CXCR5 (biotin, clone 2G8, BD Biosciences) for 45 min followed by PE-Cy7-conjugated streptavidin for 20 min.

For intracellular staining, cells were restimulated with phorbol myristate acetate (50 ng/ml, Sigma Aldrich), ionomycin (250 ng/ml, Sigma Aldrich) in the presence of monensin (1/1000, eBioscience). Cells were fixed and permeabilised with the Cytofix/Cytoperm kit (BD Biosciences) and stained with a recombinant mouse IL-21R subunit-human Fc chimaera (R&D systems, 596-MR), followed by a PE-conjugated goat anti-human antibody (Jackson ImmunoResearch, 109-116-088). Ki67 was then stained with an anti-Ki67 antibody (AF700, clone B56, BD Biosciences). The stained cells were analysed with a BD LSRFortessa (BD Biosciences) and BD FacsDiva was used for data collection, and FlowJo (FlowJo, LLC) was used for analysis. Gating strategy for Tfh cells is available in the supplementary file.

### Statistical analysis

Statistical analyses were performed using the GraphPad Prism software. Statistical significance was determined either by Mann–Whitney, Wilcoxon matched-pairs signed-rank or Kruskal-Wallis test followed by Dunn’s multiple comparison test. *p* *≥* 0.05: not significant; *p* *<* 0.05 and ≥0.01: *p *<* 0.01 and ≥0.001: **; *p* *<* 0.001 and ≥0.0001: ***; *p* *<* 0.0001: ****.

### Data availability

In vitro (RNA-Seq of RAW 264.7 cells stimulated with the AS03 adjuvant after 4 and 8 h) and in vivo (Expression data from draining lymph node and muscle of mice immunized with AS03 or PBS) transcriptomic data are available at ArrayExpress under the accession numbers E-MTAB-5962 and E-MTAB-6632, respectively.

## Electronic supplementary material

Supplemental figures
