# Supplementary material for: Activation of the endoplasmic reticulum stress sensor IRE1α by the vaccine adjuvant AS03 contributes to its immunostimulatory properties
Source: NPJ Vaccines. 2018 Jun 28;3:20. doi: 10.1038/s41541-018-0058-4 (PMC6023910; doi:10.1038/s41541-018-0058-4)

Supplementary Figures (Givord, Welsby *et al*)

Uncropped immunoblots for Figure 3C

Phospho IRE1α

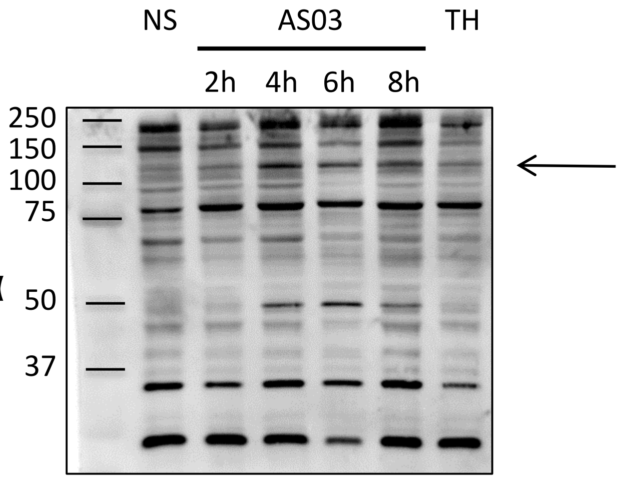

IRE1α

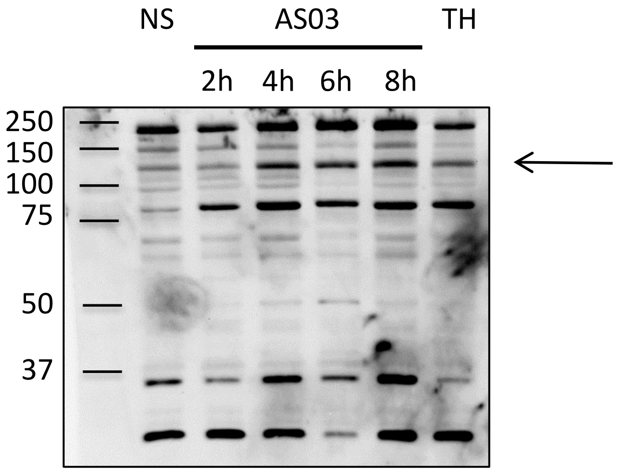

Actin

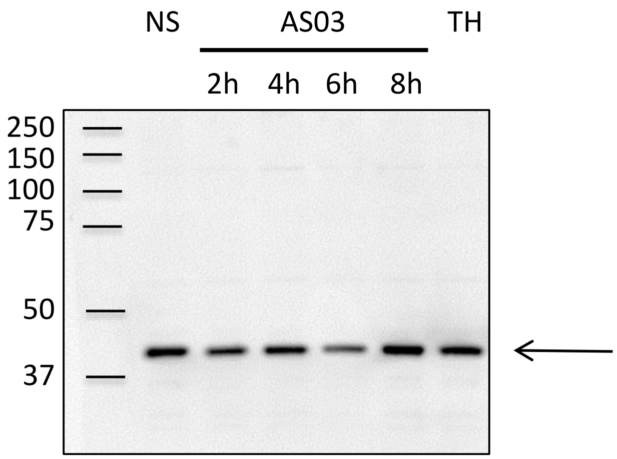

Uncropped immunoblots for Fig 3C

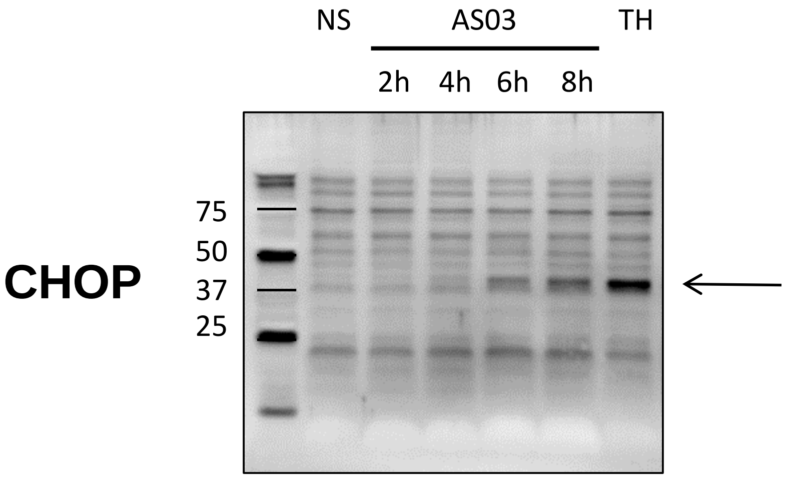

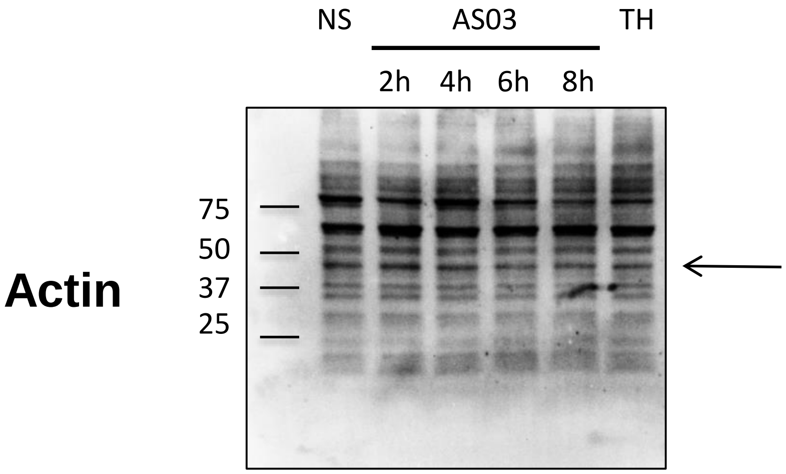

Uncropped immunoblots for Fig 3C

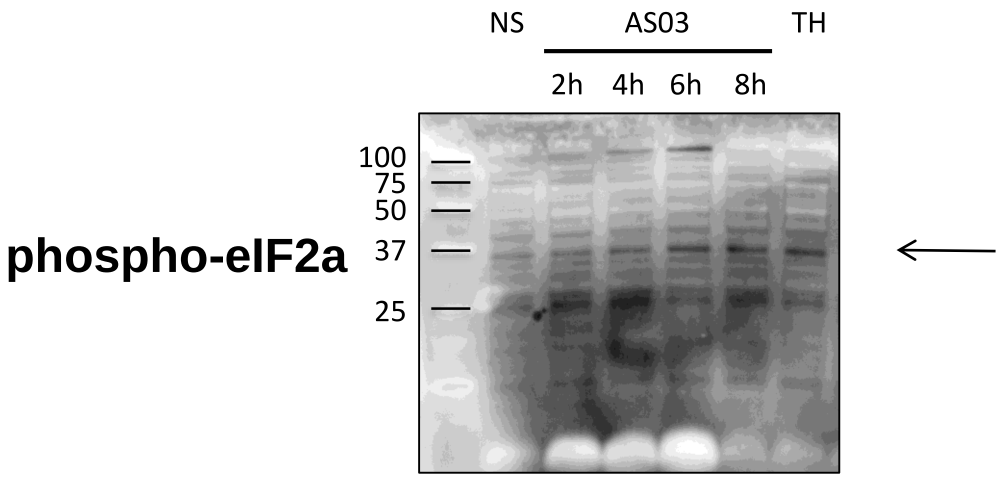

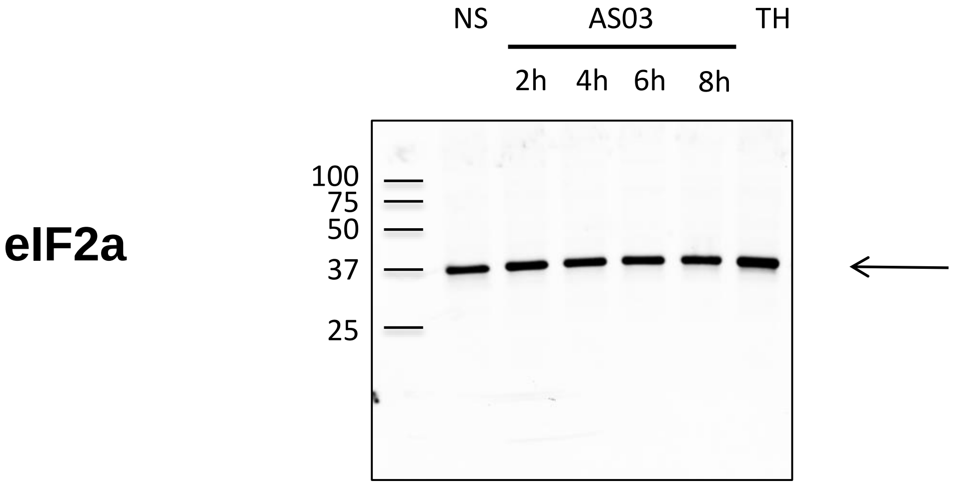

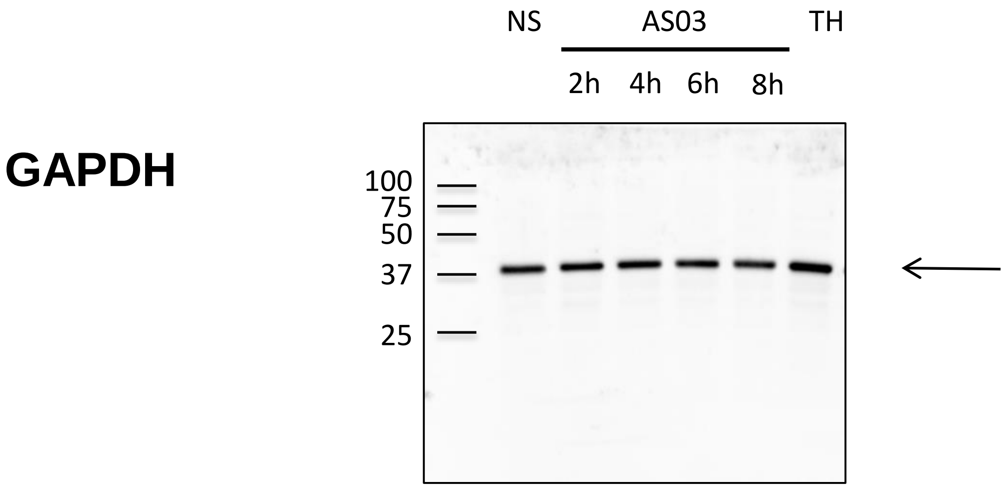

Uncropped immunoblots for Fig 3D

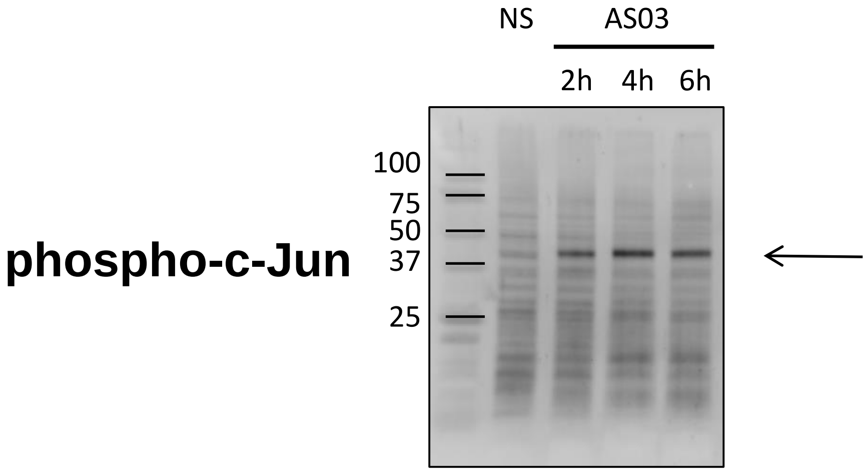

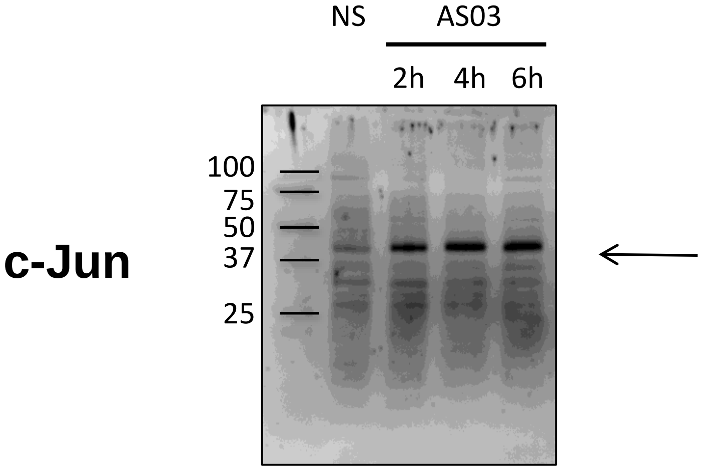

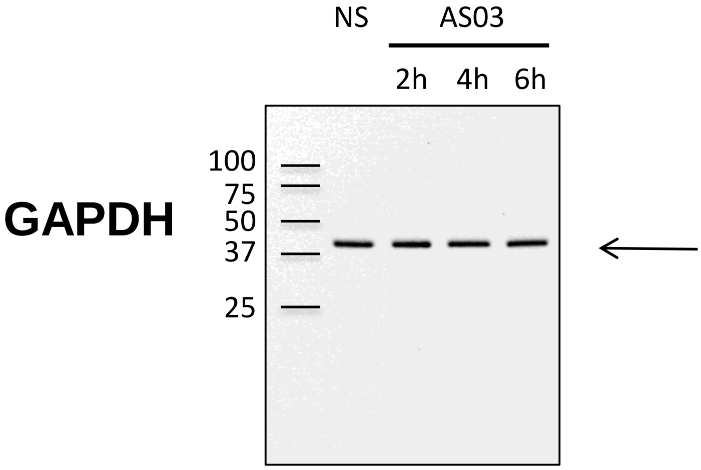

Uncropped immunoblots for Fig 4A

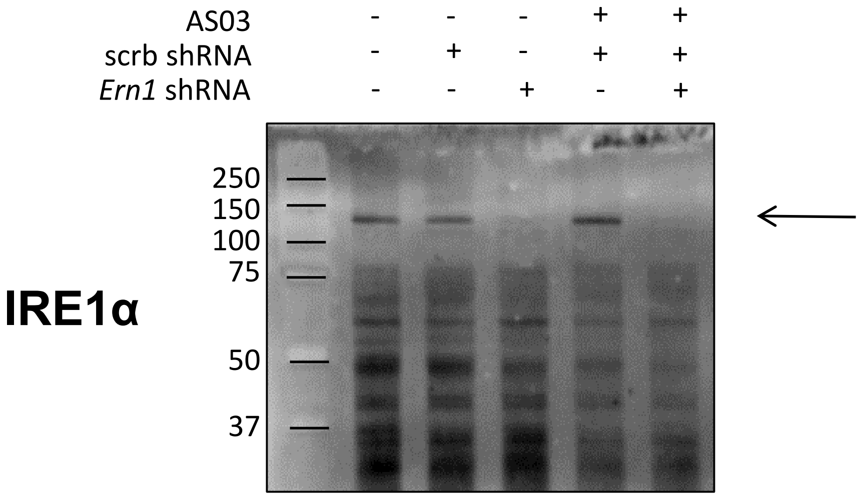

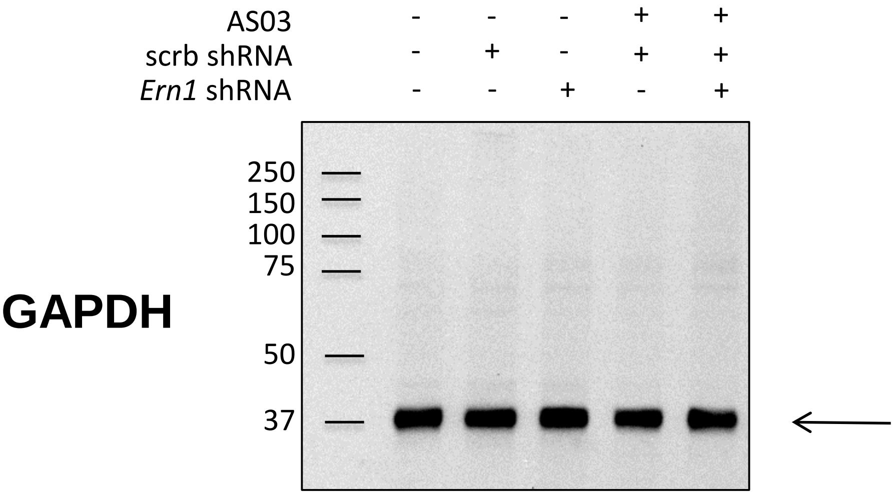

Uncropped immunoblots for Fig 4C

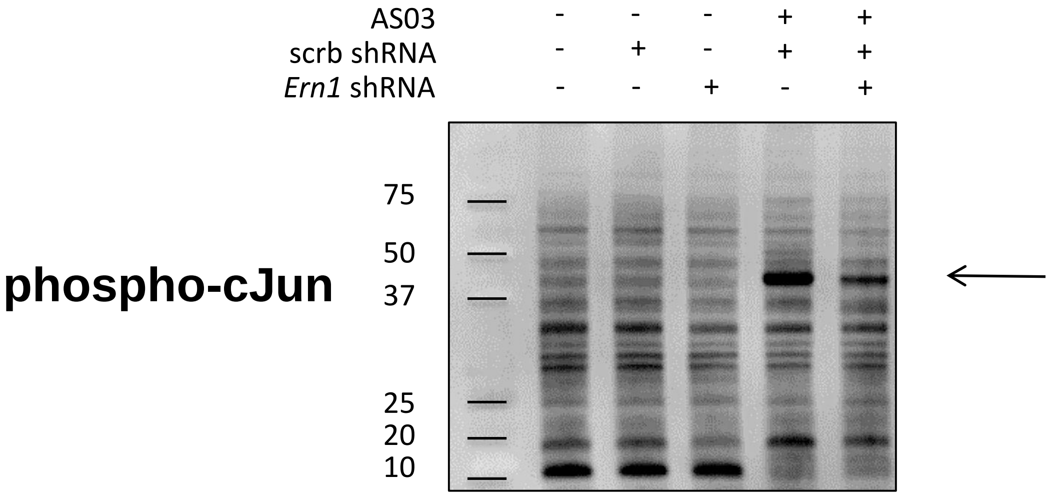

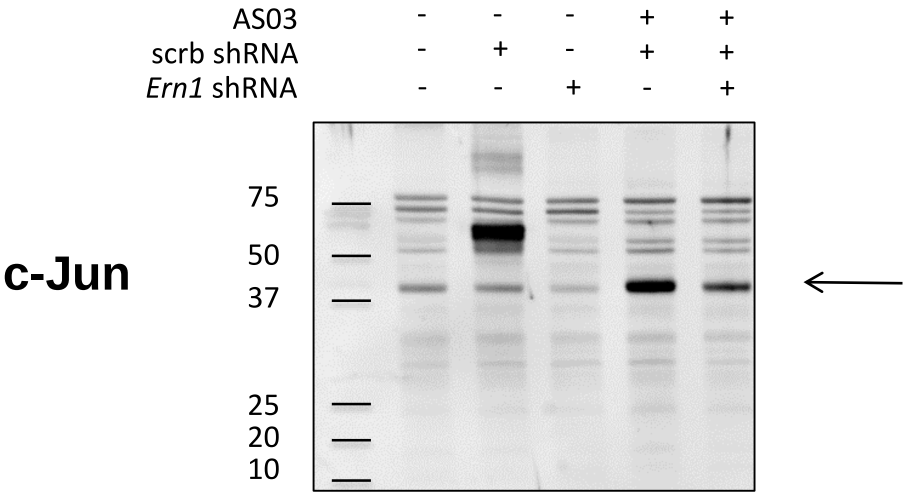

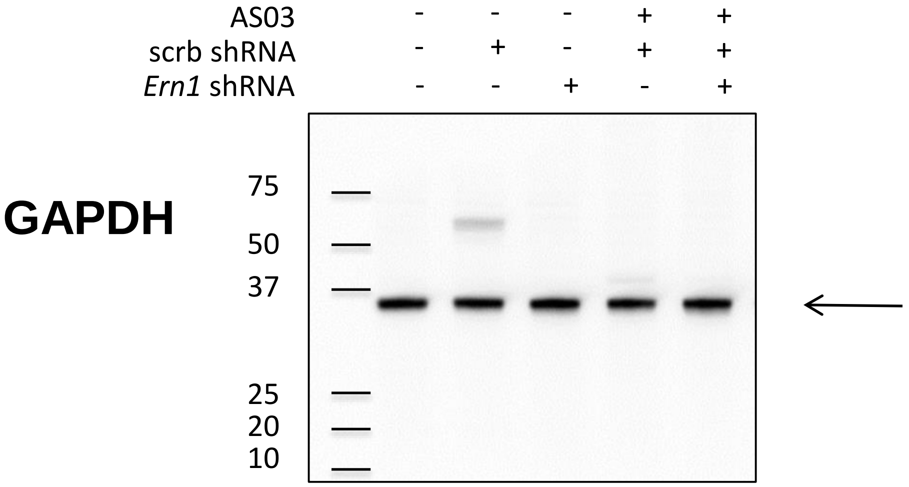

FACS Gating strategy for Tfh cells (Fig 6b)

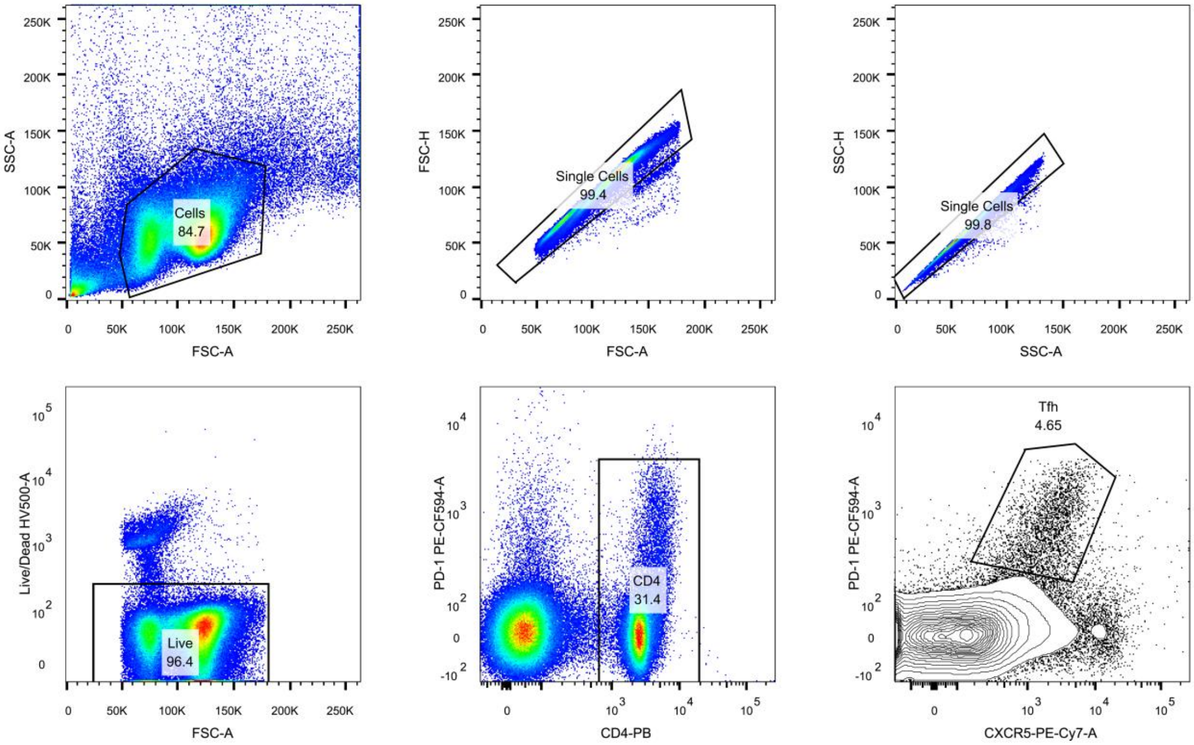

Supplement: Supplementary file 1 — Supplemental figures [file 41541_2018_58_MOESM1_ESM.pdf]
